# Brain Tumor Detection and Categorization with Segmentation of Improved Unsupervised Clustering Approach and Machine Learning Classifier

**DOI:** 10.3390/bioengineering11030266

**Published:** 2024-03-08

**Authors:** Usharani Bhimavarapu, Nalini Chintalapudi, Gopi Battineni

**Affiliations:** 1 Department of Computer Science and Engineering, Koneru Lakshmaiah Education Foundation, Vaddeswaram 522302, India; 2 Clinical Research Centre, School of Medicinal and Health Products Sciences, University of Camerino, 62032 Camerino, Italy; nalini.chintalapudi@unicam.it

**Keywords:** brain cancer, tumor detection, fuzzy c-means, MRI images, extreme learning, glioma, malignant

## Abstract

There is no doubt that brain tumors are one of the leading causes of death in the world. A biopsy is considered the most important procedure in cancer diagnosis, but it comes with drawbacks, including low sensitivity, risks during biopsy treatment, and a lengthy wait for results. Early identification provides patients with a better prognosis and reduces treatment costs. The conventional methods of identifying brain tumors are based on medical professional skills, so there is a possibility of human error. The labor-intensive nature of traditional approaches makes healthcare resources expensive. A variety of imaging methods are available to detect brain tumors, including magnetic resonance imaging (MRI) and computed tomography (CT). Medical imaging research is being advanced by computer-aided diagnostic processes that enable visualization. Using clustering, automatic tumor segmentation leads to accurate tumor detection that reduces risk and helps with effective treatment. This study proposed a better Fuzzy C-Means segmentation algorithm for MRI images. To reduce complexity, the most relevant shape, texture, and color features are selected. The improved Extreme Learning machine classifies the tumors with 98.56% accuracy, 99.14% precision, and 99.25% recall. The proposed classifier consistently demonstrates higher accuracy across all tumor classes compared to existing models. Specifically, the proposed model exhibits accuracy improvements ranging from 1.21% to 6.23% when compared to other models. This consistent enhancement in accuracy emphasizes the robust performance of the proposed classifier, suggesting its potential for more accurate and reliable brain tumor classification. The improved algorithm achieved accuracy, precision, and recall rates of 98.47%, 98.59%, and 98.74% on the Fig share dataset and 99.42%, 99.75%, and 99.28% on the Kaggle dataset, respectively, which surpasses competing algorithms, particularly in detecting glioma grades. The proposed algorithm shows an improvement in accuracy, of approximately 5.39%, in the Fig share dataset and of 6.22% in the Kaggle dataset when compared to existing models. Despite challenges, including artifacts and computational complexity, the study’s commitment to refining the technique and addressing limitations positions the improved FCM model as a noteworthy advancement in the realm of precise and efficient brain tumor identification.

## 1. Introduction

During the aging process, brain cells lose their ability to control themselves, leading to the development of brain tumors. As tumors grow in cells, they increase pressure on the brain and negatively affect overall health [1,2]. The tumors can damage normal tissues, overgrow in the brain, and replicate in other parts of the body [2,3,4]. Disease symptoms vary depending on tumor size, and the main challenge is diagnosing the type of tumor because they vary in both location and size [5]. It has been shown that early diagnosis reduces the severity and mortality of brain tumors [6].

Brain tumors, though comparatively rare in the spectrum of cancers, pose significant challenges due to their intricate location and potential impact on vital brain functions. Incidence rates vary, with an estimated annual occurrence of approximately 14 new cases per 100,000 people. According to the National Brain Tumor Society, there has been a 300% increase in brain tumor-related deaths over the past thirty years [7]. Gliomas, comprising astrocytoma’s and glioblastomas, are among the most common primary brain tumors. While overall incidence remains relatively low, the burden is substantial, given the complexities associated with diagnosis, treatment, and the potential life-altering consequences. Brain tumors exhibit a diverse age distribution, affecting individuals across the lifespan. Pediatric brain tumors are a leading cause of cancer-related mortality in children, emphasizing the urgency for specialized care and research in this population. In adults, the incidence rises with age, and certain types, such as meningiomas, are more prevalent in older individuals. Understanding age-related patterns is critical for tailoring diagnostic approaches and treatment strategies to the unique needs of distinct age groups. Identifying specific risk factors for brain tumors remains challenging, as many cases lack clear etiological markers. However, certain genetic predispositions, exposure to ionizing radiation, and hereditary conditions may elevate risk. The impact of brain tumors on quality of life is profound, as these tumors can disrupt cognitive function and sensory abilities. Symptoms vary based on tumor location and size, affecting patients’ daily lives and necessitating comprehensive care approaches that address both medical and psychosocial aspects. Untreated brain tumors can be fatal.

Diagnosing and treating brain tumors is a complex challenge for medical professionals. Prompt detection and early treatment initiation are key factors in improving survival rates for brain tumor patients [6]. Unlike biopsies for other body parts, brain tumor biopsies require surgical intervention, making them more complicated. This underscores the importance of non-surgical methods for precise diagnosis. The size and severity of a brain tumor cannot be determined by traditional images. The treatment response may be delayed due to some existing constraints. Medical professionals use magnetic resonance imaging (MRI) and computed tomography (CT) images to identify the brain’s normal and abnormal tissue [8]. Recognizing brain tumors manually is tedious and error-prone process, but automatic detection accurately identifies their regions, shape, boundaries, and position. By detecting, segmenting, and classifying brain tumors in medical images, computer-aided diagnosis can detect them [9].

Brain tumors have been identified using MRI images because they are accurate and can segment the affected area accurately. It is possible to extract accurate and highly relevant features from MRI images, which help to predict brain tumors [10]. Cluster-based segmentation divides MRI images into subcategories and indicates the region of interest (ROI) in every scan [11]. With clustering, tumor size can be reliably identified, thereby enabling effective treatment and reducing mortality risk [12]. High-similarity pixels are grouped into a separate region and separated from low-similarity pixels.

According to the American Brain Tumor Association [13], the standard classification system for brain tumors ranges from grade I to IV. Grades I and II, known as low-grade gliomas, are typically benign and grow slowly, while grades III and IV, referred to as high-grade gliomas, are malignant and grow quickly. If not treated, a low-grade brain tumor may progress to a high-grade, more dangerous form. Regular monitoring through MRI or CT scans every six months to a year is recommended for patients with grade II gliomas. Brain tumors can affect individuals of any age and have varying effects on the body.

Low-grade gliomas (grades I and II) are generally considered curable with complete surgical removal, while high-grade (grades III and IV) malignant brain tumors are treated with radiation, chemotherapy, or a combination of both. Malignant gliomas include both grade III and IV gliomas, also known as anaplastic astrocytoma’s, which are mid-grade tumors that grow more abnormally and rapidly than lower-grade tumors. The most severe form of astrocytoma, known as glioblastoma, is a grade IV tumor characterized by unusually rapid blood vessel growth and the presence of necrosis. Glioblastomas are the most aggressive and rapidly growing tumors in this classification.

Segmentation in medical imaging is crucial for identifying affected tumor tissues. It involves dividing an image into sections or segments with similar attributes like color, texture, contrast, brightness, and gray level. In brain tumor segmentation, there is a need to distinguish tumor tissues, including edema and necrotic cells, from normal brain tissues and solid tumors, like white matter (WM), gray matter (GM), and cerebrospinal fluid (CSF), using MRI or other imaging methods.

MRI images of brain tumors can be segmented using K-means clustering with high precision [14]. Based on a study that classified brain tumors, local-level features were identified [15]. Membership in the fuzzy C-Mean (FCM) is not a measure of the degree of the corresponding data. The values of each element range from 0 to 1. The improved FCM algorithm is a type of soft clustering that assigns membership levels to each data point for multiple clusters, rather than forcing a hard categorization into a single cluster. This method is particularly useful in medical imaging, where the boundaries between different tissue types, such as tumor and normal brain tissue, can be ambiguous or overlapping. In [16], the authors used the FCM approach to generate membership and typicality values for unlabeled clustering data. An FCM technique can be used to generate membership and possibility under practical point models or cluster centers [17]. Some works implemented FCM techniques, both adaptive and non-adaptive for determining local spatial element weights [18,19].

When dealing with various levels of noise, probabilistic FCM proved to be more reliable and effective. An exponential FCM clustering technique has been proposed to overcome FCM’s noise condition limitation [20]. The exact details of tumors are not identified in most existing models, and it is difficult to segment the precise location of tumors. There is difficulty identifying tumor margins and areas, and limited features can reduce classification error rates. This leads us to propose an improved FCM that is capable of detecting tiny brain tumors with high accuracy. A color and texture feature extraction approach were applied after segmentation to MRI images. To classify brain tumors, the extreme learning machine (ELM) was developed.

The proposed study makes a substantial advancement in brain tumor detection and segmentation compared to existing works by introducing an improved Fuzzy C-Means (FCM) clustering algorithm. In contrast to traditional clustering techniques, the enhanced FCM model excels in identifying minute brain tumors and showcases superior performance in accurately categorizing diverse tumor types. The algorithm’s ability to handle uncertainty, reduce variability in segmentation results, and provide a nuanced approach with degrees of membership, non-membership, and hesitation degree represents a significant breakthrough.

### Research Contributions

➢ The study contributes by utilizing the Fig share and Kaggle datasets for brain tumor detection. These datasets provide a diverse and comprehensive set of brain images, ensuring the applicability and robustness of the proposed methods across various scenarios.
➢ A novel contribution lies in the proposal of an improved fuzzy C-Means clustering algorithm. This technique specifically addresses the challenge of detecting minute tumors, showcasing an advancement in the ability to identify smaller lesions that might be overlooked by traditional methods.
➢ The research conducts an extensive investigation by evaluating the proposed improved fuzzy C-Means clustering against five state-of-the-art segmentation models. This comparative analysis contributes valuable insights into the strengths and weaknesses of different segmentation approaches, providing a basis for selecting optimal methods in brain tumor detection.
➢ The study introduces a rigorous evaluation framework by incorporating key metrics such as accuracy, precision, recall, and F1-score. These metrics offer a comprehensive assessment of the proposed approach’s performance, allowing for a nuanced understanding of its effectiveness in comparison to existing models.
➢ A significant research contribution is the demonstration of fast and accurate tumor detection achieved by the proposed approach. This highlights the practical viability of the method in real-time scenarios, contributing to the efficiency of brain tumor detection over existing state-of-the-art approaches.

## 2. Literature Review

This section discusses the literature review for brain tumor detection and classification. Cherukuri et al. [21] employed Xception as the foundational architecture and introduced a multi-level attention network (MANet) incorporating both spatial and cross-channel attention mechanisms on the 3064 T1W-CE MRI datasets, achieving an accuracy of 96.51%. An inherent drawback of long-short-term memory (LSTM) networks is their heightened computational cost and the need for intricate network tuning. LSTMs, being more intricate than CNNs and RNNs, present challenges in debugging and issue identification. Guan et al. [22] initially enhanced the visual quality of input images through contrast optimization and nonlinear strategies. Subsequently, tumour locations were determined using segmentation and clustering techniques. These locations were then utilized in conjunction with the corresponding input image, feeding into Efficient Net for feature extraction. Despite achieving an accuracy of 98.04% with fivefold cross-validation on the T1W-CE MRI dataset, this study suffers from increased computational costs due to the necessity of training multiple networks. Badža et al. [23] proposed an advanced CNN with 22 layers for classifying three tumour types (meningioma, glioma, and pituitary) in the T1W-CE MRI dataset. Employing subject-wise and record-wise tenfold cross-validation on both augmented and original image databases, they obtained the highest accuracy of 96.56% on the 3064 T1W-CE MRI datasets. Deepak et al. [24] utilized deep learning and machine learning, modifying a pre-trained Google Net with the Adam optimizer. Incorporating SVM or KNN instead of the classification layer within the transfer learning model improved accuracy to 92.3%, 97.8%, and 98% for Google Net, SVM, and KNN, respectively, with fivefold cross-validation on the T1W-CE MRI dataset. However, this research faces challenges, including the relatively poor performance of the transfer-learned model as an independent classifier and significant misclassification in meningioma class samples.

Díaz-Pernas et al. [25] proposed a method for brain tumours segmentation and classification on the T1W-CE MRI dataset using sliding window segmentation with a N window. Applying data augmentation to prevent overfitting, the classification CNN with three pathways for feature extraction achieved an accuracy of 97.3% on the T1W-CE MRI dataset. The limitation includes the lowest sensitivity for meningioma due to lower contrast intensity between the tumours and healthy areas. Alhassan et al. [26] introduced a CNN with a hard swish based RELU activation function involving image pre-processing and HOG feature descriptor utilization; this method achieved an accuracy of 98.6% for brain tumours classification on the T1W-CE MRI dataset.

Ghassemi et al. [27] employed a pre-trained deep convolutional neural network as a GAN discriminator (DCGAN) with a SoftMax layer within the GAN discriminator. They achieved an accuracy of 95.6% for brain tumours classification on the T1W-CE MRI dataset. A drawback is the limitation of GAN due to a network size of 64, preventing the use of pre-trained architectures as discriminators that require larger input sizes. Noreen et al. [28] utilized deep learning and machine learning, applying fine-tuned Inception-v3 and Xception for feature extraction and classification with SVM, KNN, random forest (RF), and ensemble techniques. The Inception-v3-Ensemble method produced the best testing accuracy of 94.34% among all proposed methods but suffered from time-consuming and high computational costs.

Gumaei et al. [29] proposed a regularized extreme learning machine (RELM) as a hybrid feature extraction method, including min–max normalization for pre-processing and PCA-NGIST for feature extraction; this method achieved an accuracy of 94.233% with fivefold cross-validation on the T1W-CE MRI dataset. Haq et al. [30] developed a DCNN technique for brain tumours detection and classification, involving pre-processing with N4ITK, normalization, and data augmentation. They used a Google Net variant model with and without conditional random fields (CRF), achieving 97.3% and 95.1% accuracy, respectively, on the T1W-CE MRI dataset. A disadvantage is the model’s unsuitability for classification tasks involving small amounts of data and challenges with erroneous information from various imaging modalities.

Ghosal et al. [31] employed a DCNN-based SE-ResNet-101 architecture, fine-tuned to fit training data, achieving an overall accuracy of 89.93% and 93.83% without and with data augmentation, respectively. Nawaz et al. [32] utilized a custom Corner Net with DenseNet-41 base network and one-stage detector, achieving 98.8% accuracy based on the T1W-CE MRI dataset, providing a low-cost solution to brain tumours classification. Verma et al. [33] suggested Hyper-Sphere Angular Deep Metric-based Learning (HSADML) with Mobile Net as the backbone network, achieving 98.69% overall accuracy based on the T1W-CE MRI dataset. The suggested method enhanced intra-class separability and decreased intra-class variability. However, the research did not emphasize the backbone network, leaving room for introducing attention-based domain-specific networks.

Cinar et al. [34] applied image cropping and various data augmentation techniques, designing a CNN from scratch for brain tumour classification. They achieved overall accuracies of 98.09%, 98.32%, and 96.35% with different training and testing dataset divisions, demonstrating categorization without relying on deep networks. A drawback is the long training period without transfer learning, making it impractical for larger datasets. Deepak et al. [35] proposed a custom CNN model, achieving accuracy improvements from 94.2% to 95.8% with SVM using a fivefold cross-validation method. Deepak et al. [36] introduced deep feature fusion and majority voting approaches, enhancing CNN predictions for three types of brain tumours but increasing computational complexity due to training with multiple loss functions.

Kumar et al. [37] employed three CNN models—Alex Net, ResNet 50, and Inception V3—resizing images, normalizing between [0] and [1], and applying augmentation techniques. They achieved accuracies of 93.51%, 98.24%, and 92.07%, respectively. Despite demonstrating the efficiency of CNN architectures in identifying enhanced MRI brain tumour images, the work has limitations, including the computational slowness of CNNs with operations like max pooling, the prolonged training time for multiple layers, and the demand for substantial training data.

Diverse methodologies and algorithms have been explored in prior works for brain tumour classification, each with its own set of limitations. Deep learning approaches, while capable of extracting features directly from input data, entail high complexity, fixed input image sizes, and costly computational requirements. The selection of an appropriate deep learning model with optimal hyperparameters remains a challenging task in previous works. To address these limitations, this research minimizes pre-processing steps and carefully chooses optimization models with suitable parameters.

## 3. Materials and Methods

**Figure 1:**
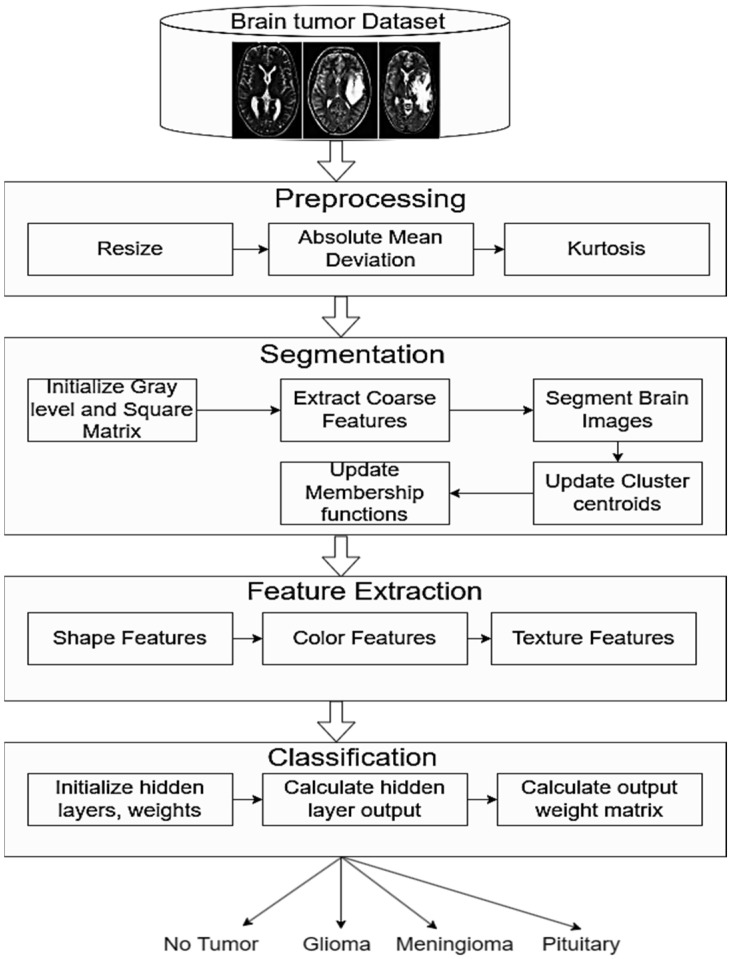
Brain tumor detection and classification flow diagram.

The proposed framework for brain tumor detection and classification is shown in Figure 1. It consists of four stages, including image preprocessing, segmentation, feature extraction, and classification, that are further explained below. We gathered brain tumor images from different datasets. In the first stage, we proposed an improved hybrid contrast enhancement based on the absolute mean deviation and the kurtosis function to enhance the contrast of the brain tumor images. In the second stage, we proposed the improved fuzzy model to segment the brain tumor images. In the third stage, the feature extraction process extracts the shapes, textures, and colors of the segmented brain tumor images. In the final stage, the enhanced ELM model correctly identified four types of brain tumors: pituitary, no tumor, meningioma, and glioma tumors.

We collected MRI images from two open-source libraries: the Fig share [38] and Kaggle datasets [39]. In Table 1, we describe the image distribution of different brain MRI tumors.

**Figure 2:**
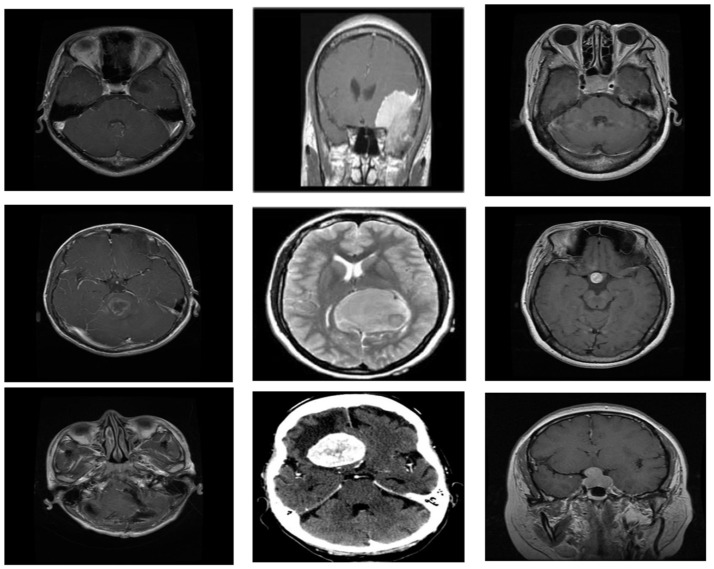
MRI image classes i.e., Glioma (**left**), Meningioma (**Middle**), and Pituitary (**Right**).

Figure 2 shows sample image classes of brain tumors. It has three subfigures, and we explain each subfigure below:

(a) Glioma:

Glioma is a category of brain tumor that originates from the glial cells, which are supportive cells in the brain. These tumors can occur in various parts of the brain and spinal cord. Gliomas are known for their diverse and aggressive nature, often infiltrating surrounding brain tissues. They are classified into different types, such as astrocytoma’s, oligodendrogliomas, and ependymomas, based on the specific glial cell they originate from. Gliomas pose challenges in treatment due to their infiltrative growth and resistance to therapies.

(b) Meningioma:

Meningioma is a type of brain tumor that arises from the meninges, the layers of tissue covering the brain and spinal cord. These tumors are generally slow-growing and often benign, but they can cause symptoms depending on their size and location. Meningiomas can develop along the meninges’ surface and may press against adjacent brain structures, leading to a range of neurological symptoms. Surgical removal is a common treatment approach for meningiomas, and they are generally associated with a favorable prognosis.

(c) Pituitary:

Pituitary tumors originate in the pituitary gland, a small gland at the base of the brain responsible for regulating various hormones. These tumors can be either benign or malignant and may affect hormone production. Depending on their size and hormone-secreting activity, pituitary tumors can lead to hormonal imbalances, impacting bodily functions. Common types include prolactinomas, growth hormone-secreting tumors, and non-functioning adenomas. Treatment options vary and may include surgery, medications, or radiation therapy, aiming to restore hormonal balance and manage symptoms.

### 3.1. Image Preprocessing

In this study, we proposed an improved hybrid contrast enhancement based on the absolute mean deviation and the kurtosis function. This approach increases the contrast of the brain tumors and makes the brain images more useful for further processing. Consider D as the brain tumor database, and N is the brain image dimensions PXQ input.

Let ∆ denote the brain tumor database and X represent an input image with dimensions N × M. Let n denote the total number of pixels in the image, and x_i_ represent each image pixel. The absolute mean deviation (AMD) and kurtosis are expressed through Equations (1) and (2).

(1) $$AD=\frac{1}{n}\sum_{i=1}^{n}\left|x_{i}-\phi \left(X\right)\right|$$

(2) $$SK=\frac{\frac{1}{s}\sum_{k=1}^{m}{\left\|x_{i}-X\right\|}^{3}}{\left(n-1\right)\;*\;{\left\|s\right\|}^{3}}$$

Here, AD represents the AMD of the image, φ(X) is the average mean of the dataset (∆), and S is the standard deviation, and it is represented as $\sqrt{\frac{\sum_{i=1}^{n}{\left(x_{i}-\bar{x}\right)}^{2}}{n}}$. $\bar{X}$ is the mean (average) of the data points. Employing these values, the final transformation of the image is $I_{1}=AD\left(i\right)+X$ and $IF=I_{1}-SK\left(i\right)$, where *IF* is the final transformed image, and i denotes the image pixels. This procedure is implemented across the entire selected datasets prior to the training of the learning models.

### 3.2. Segmentation

Segmentation is an important method for analyzing image processing, and it is crucial for diagnosing illnesses using MRI images. By doing so, digital image analysis will be made easier and more accessible. When the MRI image is segmented, it is divided into several homogeneous sections that are not overlapping. The foundation of segmentation lies in unsupervised clustering algorithms. A fuzzy model is used to address uncertainties related to borders, variations, and vagueness in gray-level images [40]. Among the methods of sorting unlabeled data into discrete sets, clustering is the most commonly used. In the present study, an output window is selected, and FCM clustering segmentation is used to segment it. Algorithm 1 presents the improved FCM algorithm after segmenting the brain tumor images.


**Algorithm 1: Improved FCM**
**Input:** Gray level image **Output:** Segmented image
➢Set the number of intensity levels and compute a square matrix; I = $\sum \sum G\left(p_{i},q_{j}\right)$, and the image matrix G = $\sum \sum G\left(p_{k},q_{l}\right)$➢Initialize $F_{xy}$ **=** G⊕I➢Extract coarse features from $F_{xy}$➢Segment the brain image$G_{1}$ **=** $\frac{1}{2}\sum_{k=1}^{m}\sum_{l=1}^{n}G_{kl}{\left\|p_{k}-a_{l}\right\|}^{2}*\sum_{k=1+1}^{X}\sum_{l=1+1}^{Y}T{\left\|p_{k},q_{l}\right\|}^{2}$➢Repeat until $F_{xy}\leq \sum_{i=1}^{H}\sum_{j=1}^{z}\left[F_{xy}\left(i\right)-F_{xy}(j)\right]$➢Update $G_{1}$➢Initialize cluster centroid➢Define membership function $s_{kl}^{\left(0\right)}$ of the FCM➢Update cluster center $T_{k}^{\left(v\right)}\Leftrightarrow s_{kl}^{\left(v\right)}$, (*k* = 1,2,… and *v* = 1,2,3)Update D($p_{k}$, $\delta_{k}^{\left(v\right)}$) $\Leftrightarrow \delta_{k}^{v}$ and $s_{kl}$➢With c($p_{k}$, $\delta_{k}^{\left(v\right)}$) until ||$s_{kl}^{\left(v\right)}-s_{kl}^{\left(v+1\right)}$|| ≤ ∈, ∈ =[0,1]


The algorithm begins by setting the number of intensity levels and computing a square matrix I = $\sum \sum G\left(p_{i},q_{j}\right)$, where the image matrix *G* = $\sum \sum G\left(p_{k},q_{l}\right)$ is defined. An initial feature matrix, $F_{xy}$ = G⊕I, is created, followed by the extraction of coarse features from $F_{xy}$. Subsequently, the brain image is segmented using a process involving a mathematical expression denoted as $G_{1}$. This expression involves the computation of weighted distances between elements of *G* and specific points, followed by a thresholding operation. The algorithm iteratively refines $F_{xy}$ until a certain condition is met, updating $G_{1}$ in the process. The algorithm introduces a novel approach by incorporating a cluster-centric strategy. It initializes cluster centroids, defines a membership function $s_{kl}^{\left(0\right)}$ using Fuzzy C-Means (FCM), and subsequently updates the cluster centers $T_{k}^{\left(v\right)}$ based on the membership function values. An additional update involves adjusting a variable D($p_{k}$, $\delta_{k}^{\left(v\right)}$) based on certain criteria, refining the clustering process until a convergence condition is satisfied. The variable *G* plays a crucial role in representing the original image matrix. The novelty of the algorithm lies in its integration of fuzzy clustering, weighted distance computations, and iterative refinement processes. This multifaceted approach enhances the algorithm’s adaptability to complex image characteristics and its ability to capture subtle variations in the data. The iterative nature of the algorithm, along with the incorporation of cluster-based strategies, contributes to its effectiveness in image segmentation tasks.

The algorithm’s advantages stem from its incorporation of weighted distance computation, iterative refinement, cluster-centric strategy, dynamic thresholding, and the utilization of the parameter G. The algorithm introduces a weighted distance computation process $G_{1}$, where distances between elements of the image matrix G and specific points are weighted based on certain criteria. This enables the algorithm to capture more nuanced relationships within the data, allowing for a more accurate representation of the underlying structures in the images. Unlike standard FCM, the proposed algorithm involves an iterative refinement process for the feature matrix $F_{xy}$. This iterative nature allows the algorithm to adapt and fine-tune its segmentation results over multiple iterations, potentially leading to improved convergence and more precise segmentation. The algorithm incorporates a cluster-centric strategy by initializing cluster centroids and updating them based on the membership functions derived from FCM. This strategy enhances the algorithm’s ability to identify and characterize different clusters within the image, resulting in more accurate and meaningful segmentation. The iterative process involves dynamic thresholding conditions, providing adaptability to varying complexities in image data. This dynamic nature allows the algorithm to handle diverse image characteristics and improve its performance in scenarios where standard FCM may struggle. The use of the variable G as the original image matrix introduces a unique aspect to the algorithm. This variable, representing the initial state of the image, contributes to the algorithm’s ability to capture and preserve essential features during the segmentation process. The convergence criteria involving D ($p_{k}$, $\delta_{k}^{\left(v\right)}$) and cluster similarity add an additional layer of refinement, potentially preventing the algorithm from converging to suboptimal solutions. This results in more reliable and accurate segmentation outcomes.

### 3.3. Feature Extraction

Feature extraction is fundamental for classification. In this study, we extract the shapes, textures, and colors that are important in representing brain tumor images. The process of extracting optimal features from brain images is challenging [41]. In feature extraction, raw data are transformed into numerical data while retaining the original information. Automated or manual models can be used to extract features. Automated feature extraction extracts only issue-related significant features, while manual feature extraction extracts all significant features. Gray-level co-occurrence matrix (GLCM) features are extracted in the present study. Measures are extracted from the GLCM functions based on the texture of the brain tumor image. Matrix dimensions define the relative frequency of pixels in a brain tumor image. Homogeneity, dissimilarity, energy, and contrast are the measures determined by the features.

Traditional feature extraction techniques such as texture, shape, and color-based methods offer distinct advantages over deep learning approaches for brain image analysis. These methods provide interpretable features, making it easier for researchers and clinicians to understand the underlying characteristics related to specific pathological conditions. Additionally, traditional techniques often perform well in scenarios with limited data, where deep learning models may struggle to generalize effectively. They are computationally efficient, making them suitable for resource-constrained environments and real-time processing requirements. The integration of domain-specific knowledge is facilitated, enhancing performance in medical imaging where expertise plays a critical role. Traditional feature extraction methods offer reduced sensitivity to data quality variations, explicit control over feature selection, transparent decision-making processes, and proven transferability across different imaging modalities. While deep learning has achieved notable success, the advantages of traditional feature extraction techniques make them valuable, especially when interpretability, efficiency, and expert control are paramount considerations in brain image feature extraction. Table 2 explains the features of brain tumor detection with definition.

### 3.4. Feature Selection

Feature selection improves classification accuracy by removing irrelevant and redundant features and selecting robust features to boost performance. This study uses the entropy-based controlled approach to choose the best features. This approach removes irrelevant features by selecting the highly robust features. Entropy for the feature vector V(P) for the M X N dimensions is represented as

(3) $$\sum_{d_{1}}\sum_{dz}min\left(d_{1},d_{2}\right)*\left[-\sum_{k=1}^{z}p_{k}log(p_{k}\right)]$$

where $d_{1},d_{2}$ are the current and the previous distance of the selected features and $p_{k}$ is the probability of the kth feature. To find the more robust features, we apply the threshold function to select the features greater than the high probability feature $T_{k}$.

(4) $$\mathrm{Final}\;\mathrm{Features}=\left\{\begin{array}{c} p\left(k\right);T_{k}\leq \sum_{k=1}^{z}p_{k}log(p_{k})\\ 0;Ohterwise \end{array}\right.$$

### 3.5. Classification

In this study, we used the ELM [42] to classify brain tumors. If the ELM has a z sample ($i_{k},o_{k}$), the output with zero errors is mathematically represented as

(5) $$o_{k}=\sqrt{\sum_{k=1}^{H}\alpha_{k}t\left(w_{k}i_{k}+b_{k}\right)},\;where\;k=1,\ldots .z$$

where $i_{k}$ and $o_{k}$ are the input and the output samples, respectively, the activation function is represented as t (.), and $w_{k\;}and\;b_{k}$ refer to the weights and the bias, respectively. $\alpha_{k}$ is a weight coefficient for the k^th^ term. 

O = Hα; where the output is represented as O = ($o_{1},o_{2},\ldots o_{n}$) and the matrix weights are represented as α = ($\alpha_{1},\alpha_{2}\ldots \alpha_{m}$). The hidden layers can be represented as

(6) $$H=\left[\begin{array}{c} t\left(w_{1}i_{1}+b_{1}\right)------t\left(w_{n}i_{1}+b_{n}\right)\\ ------------------\\ -----------------\\ ------------------\\ t\left(w_{1}i_{m}+b_{1}\right)------t\left(w_{n}i_{m}+b_{n}\right) \end{array}\right]$$

The hidden layer nodes should be less than the sample count. Equation (6) represents the structure of a single-hidden-layer Extreme Learning Machine (ELM) neural network used in the classification of brain tumors. In this equation, H denotes the hidden layer, which consists of nodes with activation functions denoted by $t\left(\cdot \right).$ Each node in the hidden layer is associated with weights $w_{ki}$ and biases $b_{k},$ where i ranges from 1 to m, representing the input features. The hidden layer’s output O is obtained by summing the product of each node’s activation function and its corresponding weight, followed by the application of the activation function $t\left(\cdot \right)$. This process is mathematically expressed as O=Hα, where $O=\left(o_{1},o_{2},\ldots ,o_{n}\right)$ represents the output vector and $\alpha =\left(\alpha_{1},\alpha_{2},\ldots ,\alpha_{m}\right)$ represents the weight matrix. The structure of the hidden layer is further detailed in Equation (6), where H is represented as a concatenation of n nodes. Each node’s activation is determined by the weighted sum of the input features $i,\left(i_{1},i_{2},\ldots ,i_{m}\right)$, with weights $w_{ki}$ and biases $b_{k}$ for each node. The activation function $t\left(\cdot \right)$ introduces non-linearity to the model, allowing it to capture complex patterns and relationships within the input data. It is worth noting that the number of nodes in the hidden layer n should be chosen judiciously, typically less than the number of samples, to prevent overfitting. The weights and biases are learned during the training process, enabling the network to map input features to the desired output for effective brain tumor classification.

### 3.6. Performance Evaluation

The performance metrics can help measure the model presented in terms of the different parameters mentioned below [43,44,45,46,47,48,49]. For instance,

In the context of brain tumor classification:

➢ Accuracy in brain tumor classification represents the overall correctness of the model in predicting different types of brain tumors.
  $$\mathrm{Accuracy}=\frac{\mathrm{Correctly}\;\mathrm{classified}\;\mathrm{brain}\;\mathrm{tumor}\;\mathrm{samples}}{\mathrm{Total}\;\mathrm{brain}\;\mathrm{tumor}\;\mathrm{samples}}$$
➢ Precision in brain tumor classification measures the accuracy of the model in correctly identifying a specific type of tumor among the predicted positive cases.
  $$\mathrm{Precision}=\frac{\mathrm{True}\;\mathrm{positives}\;\mathrm{for}\;a\;\mathrm{specific}\;\mathrm{brain}\;\mathrm{tumor}\;\mathrm{type}}{\mathrm{True}\;\mathrm{positives}+\mathrm{False}\;\mathrm{positives}\;\mathrm{for}\;\mathrm{that}\;\mathrm{brain}\;\mathrm{tumor}\;\mathrm{type}}$$
➢ Recall in brain tumor classification assesses the ability of the model to correctly identify all instances of a particular brain tumor type among the actual positive cases.
  $$Recall=\frac{\mathrm{True}\;\mathrm{Positives}\;\mathrm{for}\;a\;\mathrm{Specific}\;\mathrm{Brain}\;\mathrm{Tumor}\;\mathrm{Type}}{\mathrm{True}\;\mathrm{Positives}+\mathrm{False}\;\mathrm{Negatives}\;\mathrm{for}\;\mathrm{that}\;\mathrm{Brain}\;\mathrm{Tumor}\;\mathrm{Type}}$$
➢ F1 score in brain tumor classification provides a balance between the precision and recall, giving an overall measure of the model’s effectiveness.
  $$F1\;\mathrm{Score}=2\times \frac{\mathrm{Precision}\times \mathrm{Recall}}{\mathrm{Precision}+\mathrm{Recall}}$$

## 4. Results

In this section, we analyze the performance of the proposed model. To extract MRI image information for this study, we first used an improved hybrid contrast enhancement to improve the brain MRI image and extract visual information.

### 4.1. Segmentation Evaluation

**Figure 3:**
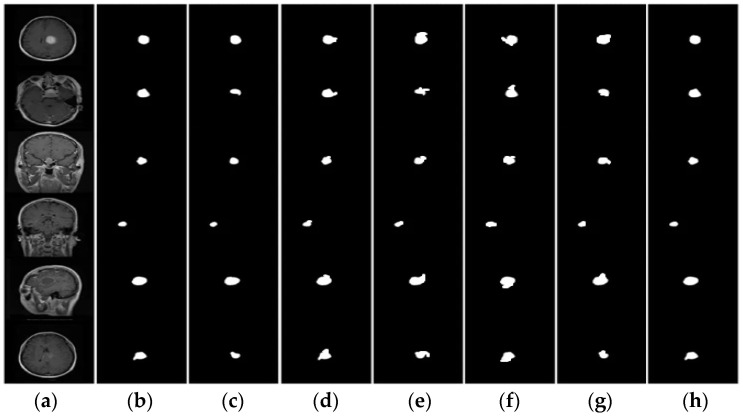
Segmentation Comparison (**a**) Original (**b**) ground truth (**c**) MLT (**d**) K-means (**e**) IPFCM (**f**) PSO (**g**) GTV_CUT_ (**h**) proposed.

The proposed model produces segmentation results that are more precise and reliable. The various segmentation results and comparisons are presented in Figure 3. The columns (a) and (b) are the original images and their corresponding ground truths. The (c–g) columns are the images implemented with the state-of-the-art approaches MLT, K-Means, IPFCM, PSO, and GTV_CUT_, respectively. The (h) column, which is implemented with the proposed approach, is similar to the ground truth MRI images.

**Figure 4:**
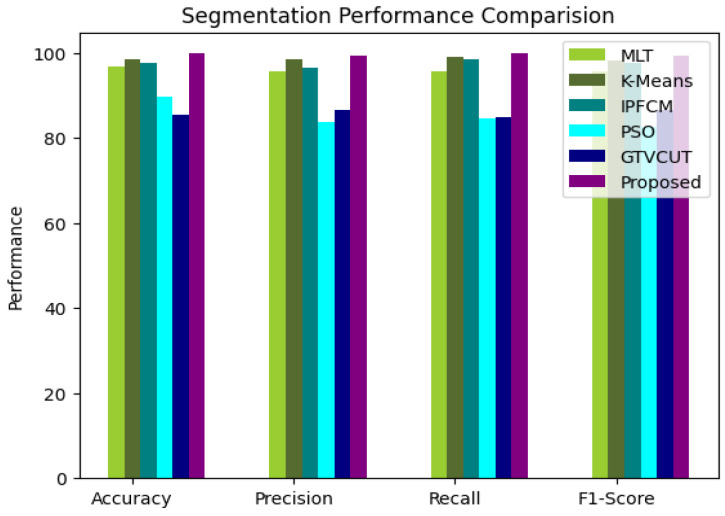
Segmentation performance comparison.

Figure 4 shows the proposed segmentation’s performance comparison with the existing models. Multilevel threshold technique (MLT) [50] segments brain tumor images and achieves an accuracy of 91.78%, precision of 90.38%, and recall of 92.75%. K-Means [51] segments brain tumor images and achieves an accuracy of 92.53%, precision of 94.57%, and recall of 94.99%. Intuitionistic possibilistic fuzzy C-Means (IPFCM) [52] segments brain tumor images and achieves an accuracy of 91.47%, a precision of 96.35%, and recall of 93.56%. Particle swarm Optimization (PSO) [53] segments brain tumor images and achieves an accuracy of 89.55%, a precision of 83.73%, and a recall of 84.57%. Gross tumor volume Segmentation (GTVCUT) [54]. The segmentation performance metrics accuracy, precision, and recall using the proposed function were 99.78%, 99.36%, and 99.75%, respectively.

### 4.2. Classification Evaluation

**Figure 5:**
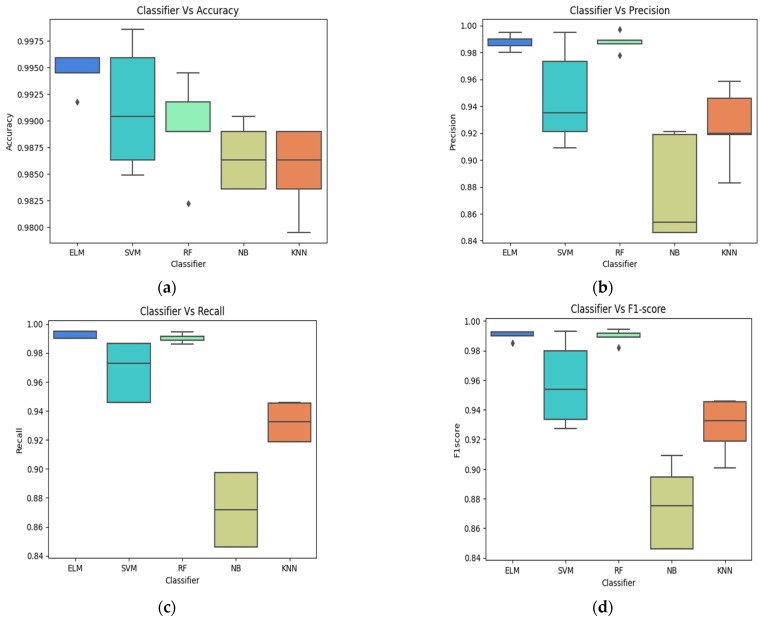
Different classifier performance in terms of accuracy (**a**), precision (**b**), recall (**c**) and F1 scores (**d**).

Figure 5 shows the performance of various classifiers on Fig share datasets brain test set images. The performance of various classifiers, namely Extreme Learning Machine (ELM), Support Vector Machine (SVM), Random Forest (RF), Naive Bayes (NB), and k-Nearest Neighbors (KNN), was evaluated on a brain tumor dataset for tumor classification across four different classes: No Tumor, Glioma, Meningioma, and Pituitary. In terms of accuracy, ELM demonstrated the highest accuracy across all classes, ranging from 97.43% to 99.25%, showcasing its robust overall performance. SVM and RF also exhibited competitive accuracy, maintaining values above 97% for most classes. KNN, while performing well, had slightly lower accuracy, especially in the Meningioma class. Figure 5a shows the classifier performance in terms of accuracy. Precision measures the ratio of correctly predicted positive observations to the total predicted positives. ELM achieved high precision values, indicating its proficiency in minimizing false positives. SVM and RF showed commendable precision as well, particularly in Glioma and Meningioma classes. KNN exhibited a slightly lower precision, especially in the Meningioma category. Figure 5b shows the classifier performance in terms of precision. Recall, or sensitivity, gauges the ability of a classifier to capture all the positive instances. ELM and SVM displayed excellent recall across all classes, ensuring comprehensive identification of positive cases. RF, NB, and KNN demonstrated reasonable recall values, with KNN exhibiting a slight dip, particularly in the Meningioma class. Figure 5c,d shows the classifier performance in terms of recall and F1 scores. 

**Figure 6:**
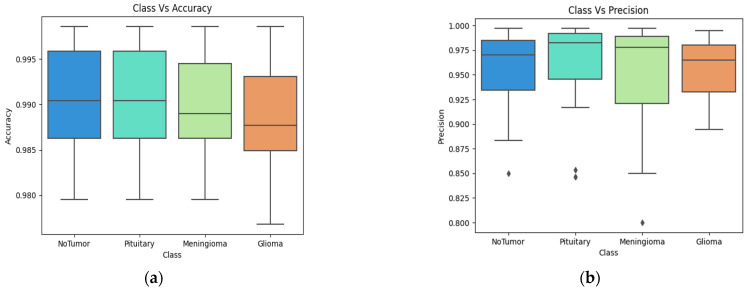

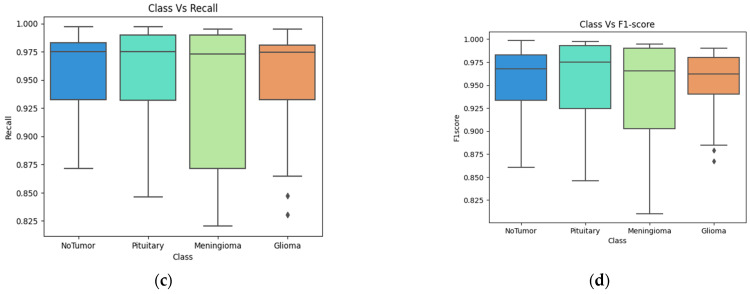
Brain tumor 4-class performance in terms of accuracy (**a**), precision (**b**), recall (**c**) and F1-score (**d**).

Figure 6 shows the class-based performance. The classification performance across the classes No Tumor, Glioma, Meningioma, and Pituitary was evaluated based on accuracy, precision, and recall metrics. In the No Tumor class, the classifiers demonstrated high accuracy, precision, and recall, with values ranging from 92.45% to 98.64%, 95.35% to 99.14%, and 90.35% to 99.25%, respectively. For the Glioma class, consistent accuracy rates were observed, ranging from 88.45% to 98.66%, precision values varied between 88.45% and 99.14%, and recall rates were between 89.74% and 99.19%. In the Meningioma class, the classifiers exhibited robust accuracy, precision, and recall percentages, with values spanning 94.57% to 98.50%, 92.10% to 98.62%, and 89.74% to 99.16%, respectively. The Pituitary class also showcased commendable performance metrics, with accuracy ranging from 94.59% to 98.64%, precision varying between 94.59% and 99.15%, and recall rates spanning 91.42% to 97.43%. Overall, the classifiers demonstrated consistent and effective performance in accurately classifying instances across diverse tumor categories, emphasizing their potential suitability for medical imaging applications. The class performance in terms of accuracy, precision, recall and F1-score in shown in Figure 6a–d.

**Figure 7:**
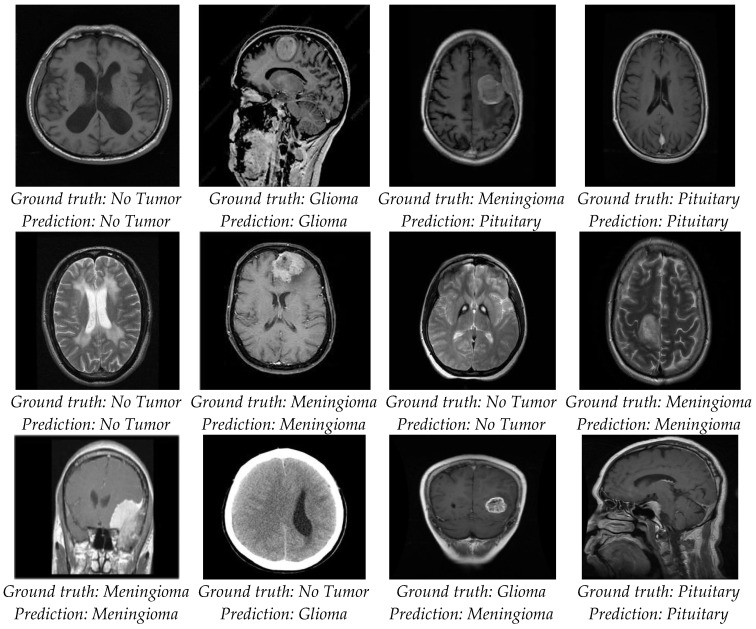
Prediction of brain tumor test images.

The results revealed that the overall testing accuracy and ELM performance metrics were most appropriate for detecting brain tumors. In total, 96.6% of the predictions were correct based on the prediction output of the ELM. ELM’s prediction output reflects the probability of correct predictions and their accuracy. The ground truth and ELM model predictions for the test images are shown in Figure 7. Ground truth is represented by the first row in the image, while predicted results are represented by the second row.

Both the generalization efficiency and the ELM rate of learning are higher than those of traditional state-of-the-art classifiers. When it comes to the ELM algorithm, weights and hidden biases are assigned regularly.

The proposed classifier demonstrates exceptional performance across multiple metrics, achieving an overall accuracy of 98.56%. For the “No Tumor” class, the classifier exhibits high precision (99.14%) and recall (99.25%). In the case of Glioma, the precision and recall stand at 98.66% and 99.19%, respectively. Similarly, for Meningioma, the classifier attains a precision of 99.14% and a recall of 99.10%. The Pituitary class also sees impressive results with a precision of 97.43% and recall of 97.43%. In comparison, the approach by Alanazi et al. [55] yields slightly lower accuracy at 97.86%. The “No Tumor” class exhibits precision and recall values of 98.49% and 98.33%, respectively. Glioma achieves a precision of 98.17% and a recall of 99.14%, Meningioma records a precision of 97.33% and a recall of 98.64%, and Pituitary shows a precision of 98.51% and a recall of 99.15%.

**Figure 8:**
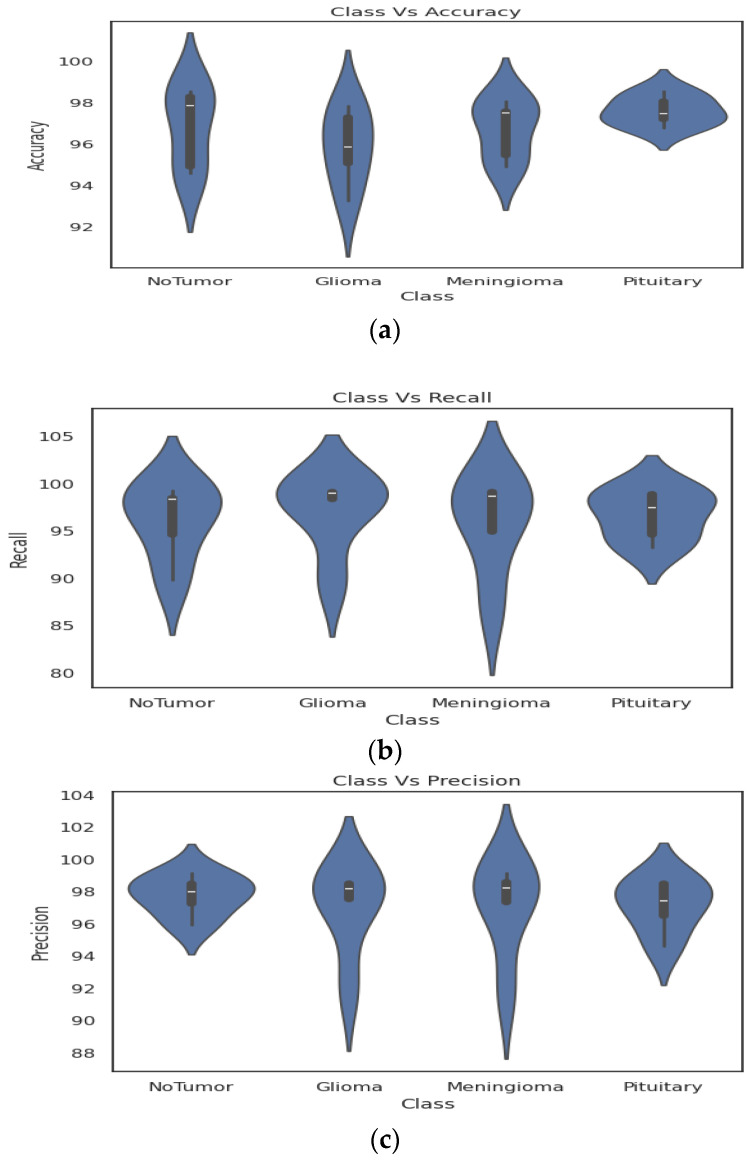
Visualization of results (**a**) Class Vs Accuracy (**b**) Class Vs Recall (**c**) Class Vs Precision.

The model developed by Zahoor et al. [56] reports an accuracy of 98.31%. However, there is a notable decrease in recall for the “No Tumor” class at 89.74%. Glioma maintains a precision of 98.49% and a recall of 98.3%, while Meningioma achieves a precision of 98.62% and a recall of 99.16%. Pituitary records a precision of 94.59%. The classifier developed by Ait Amou et al. [57] exhibits an accuracy of 94.57%. The “No Tumor” class shows a precision of 98.00% and recall of 98.50%. Glioma, however, experiences a decrease in recall (89.74%). Meningioma maintains a precision of 98.24% and a recall of 94.91%, and Pituitary shows a precision of 98.61% and a recall of 98.89%. Lastly, the model developed by Kirbayi et al. [58] demonstrates an accuracy of 94.89%. The “No Tumor” class achieves a precision of 95.90% and a recall of 94.59%. Glioma records a precision of 97.51% and a recall of 98.98%. Meningioma exhibits a precision of 91.89% and a recall of 87.17%, while Pituitary achieves a precision of 96.49% and a recall of 93.22%. Figure 8 shows the visualization results of Table 3.

Table 4 tabulates the performance comparison of the proposed with the state-of-the-art models on the Kaggle dataset.

**Figure 9:**
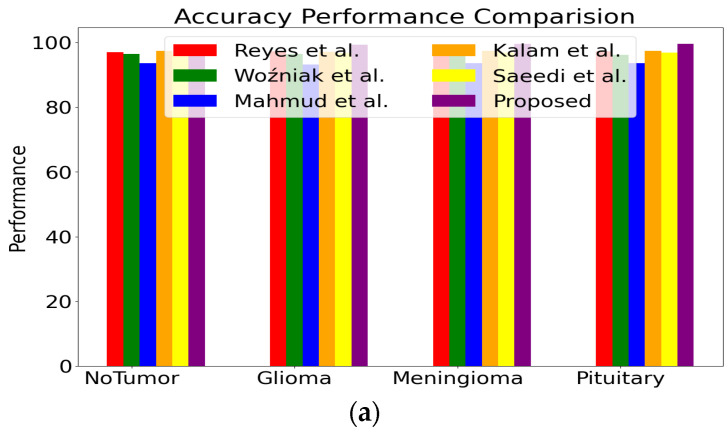

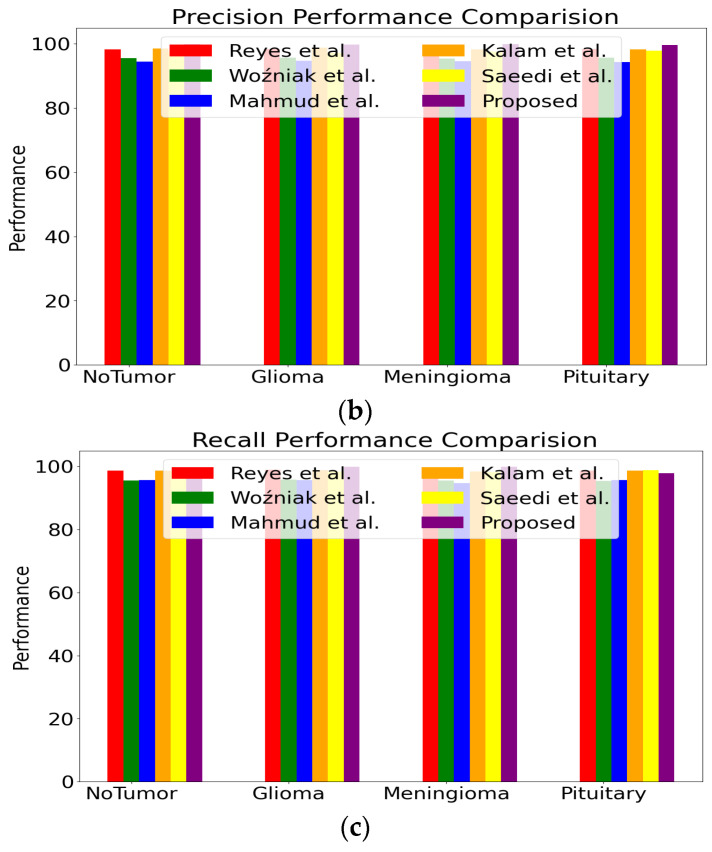
Performance Comparison (**a**) Accuracy (**b**) Precision (**c**) Recall. (i.e., Saeedi et al. [41], Kalam et al. [42], and Mahmud et al. [62], Wozniak et al. [63], and Reyes et al. [64]).

Figure 9 shows the performance comparison of the various performance metrics, such as accuracy, precision, and recall, for four classes between the proposed and the existing models [41,42,62,63,64]. In the evaluation of various classifiers for brain tumor classification, the proposed classifier demonstrates commendable performance across multiple metrics. The proposed model achieves an overall accuracy of 99.37%, showcasing its robust ability to correctly classify instances. Precision and recall values further underscore its efficacy, with 99.64% precision and 99.57% recall for the “No Tumor” class, 99.74% precision and 99.85% recall for “Glioma”, 99.85% precision and 99.84% recall for “Meningioma”, and 99.57%, 99.85% precision and recall for “Pituitary”.

Comparing this proposed classifier with existing studies, Saeedi et al. [41] achieved an accuracy of 96.46%, demonstrating slightly lower performance than the proposed model. Kalam et al. [42] and Mahmud et al. [62] also reported accuracies of 98.35% and 93.53%, respectively, placing them in line with or slightly below the proposed classifier. Woźniak et al. [63] obtained an accuracy of 96.46%, and Reyes et al. [64] achieved an accuracy of 98.03%. These comparative studies highlight the competitive nature of the proposed classifier, positioning it favorably in the landscape of brain tumor classification algorithms.

In the comparison of precision and recall values across various classifiers for brain tumor classification, the proposed classifier demonstrates notable performance. For the “No Tumor” class, the proposed model achieves precision and recall values of 99.14% and 99.25%, respectively. In the “Glioma” class, precision is reported at 98.66%, with a corresponding recall of 99.19%. The “Meningioma” class showcases precision and recall values of 99.14% and 99.10%, respectively, while the “Pituitary” class reports precision and recall of 97.43%.

Comparatively, Saeedi et al. report precision and recall values ranging from 97.57% to 98.73%, Kalam et al. present precision and recall values in the range of 98.15% to 98.74%, and Mahmud et al. show precision and recall values from 94.36% to 95.63%. Woźniak et al. and Reyes et al. also report precision and recall values in the mid-to-high 90s range.

Overall, the proposed model not only achieves impressive accuracy but also excels in precision and recall, showcasing its potential as a robust and reliable tool for brain tumor classification. This comparative analysis underscores the effectiveness of the proposed classifier in achieving a balanced performance between precision and recall, essential for reliable and accurate brain tumor classification.

## 5. Discussion

Today, brain tumors are among the most serious diseases, and their incidence is increasing. Since brain tumors can be dangerous and can only be prevented at an early stage by total brain area scanning to detect the tumor; as such, accurate diagnosis and necessary patient treatment are crucial. It is challenging to calculate the area, determine the degree of uncertainty in the segmentation area, and segment the tumor due to the structural complexity and unpredictability of brain tumors, high volatility, and intrinsic features of MRI data, i.e., variability of tumor size and shape. Medical professionals may occasionally notice variations in segmentation results due to variations in tumor form and shape. While some tumors, like meningiomas, are easy to separate, others, like gliomas, are more challenging. Meningioma and pituitary have similar textures and intensities, making it difficult to distinguish them. As a result, producing manual tumor segmentation is a laborious task.

It is essential to create an automatic detection system that can provide quick, inexpensive, accurate, and comprehensible diagnoses to handle massive amounts of data and minimize human error during screening. The workload of medical personnel is lessened by computer-assisted diagnostics, which also produces faster, more precise diagnoses with fewer mistakes. Thus, it is anticipated that clinical decision-making by physicians will be aided by computer-assisted diagnostics. Furthermore, with the aid of computer-assisted diagnostics, new avenues for research in clinical tests may become available.

Deep learning techniques acquire knowledge by directly obtaining features from images. Modern deep learning techniques, particularly CNNs, are very accurate and widely used in medical image analysis, including MRI analysis. These techniques have several drawbacks, including the need for a sizable training dataset, a high degree of temporal complexity, poor accuracy for applications with limited datasets, and a high initial cost to the user due to the requirement for expensive GPUs. It can also be quite difficult to select the most accurate deep-learning model because it involves understanding a wide range of parameters, training strategies, and topologies.

Existing models may struggle to detect tiny brain tumors, highlighting a potential drawback in their ability to handle smaller lesions [65]. Some methods, like the one utilizing borderline pixel detection, may not accurately identify the inner borders of tumor regions, impacting the completeness of segmentation [66]. The complexity of certain models, such as the Link Net network, might make them challenging to implement and interpret [67]. The application of advanced models, like 3D convolution architectures, may require substantial computational resources [68]. The overfitting problem was addressed by recommending a brain tumor method based on the ResNet50 model and global average pooling, but potential limitations remain [69]. Despite the success of certain methods, the study acknowledges the need for further research, optimization, and improvements to address limitations in accuracy and processing time [70,71,72]. The existing methods were often evaluated on specific datasets, and their generalizability to diverse datasets or clinical settings may need validation. This paper makes substantial contributions by leveraging diverse datasets, introducing an innovative clustering algorithm, conducting a thorough comparative analysis with existing models, employing comprehensive evaluation metrics, and demonstrating both speed and accuracy improvements in brain tumor detection.

We have proposed an improved segmentation algorithm based on FCM to detect brain tumors from MRI images. We compare the enhanced FCM model with segmentation models based on data from Fig share and Kaggle. An improved FCM is shown to be effective in identifying brain tumors from MRI scans in the study. The model correctly identified pituitary, no tumor, meningioma, and glioma tumors as the four types of brain tumors in the fig share dataset.

A variety of clustering techniques are presented, including FCM, K-mean, MLT, PSO, and enhanced versions of these techniques. The problem of designing a thresholding technique solution is complex and requires ongoing research by medical imaging researchers. In K-mean clustering, all the data are grouped into a single, functional cluster that does not apply to all tenders. To preserve each pixel across clusters with different membership levels, FCM assigns each pixel to unlabeled fuzzy clusters. As a result of this research, it is found that the FCM’s degrees of data cannot be reflected in its membership. In medical image segmentation, FCM clustering has been demonstrated to help diagnose diseases such as brain tumors and eliminate unknown noise and uncertainty.

In this study, an enhanced version of the FCM clustering algorithm is employed to detect minuscule brain tumors, which are often challenging to identify using conventional methods. This advanced approach involves selecting a specific output window within the imaging data for analysis. The improved FCM clustering segmentation is then applied to this targeted area. By using improved FCM clustering segmentation on the chosen output window, the algorithm effectively reduces uncertainty and increases the accuracy of tumor detection. This improvement is crucial in the context of brain tumor identification, as it allows for more precise delineation of tumor boundaries and enhances the detection of smaller, less distinct tumors. The enhanced FCM approach is instrumental in improving diagnostic accuracy, which is vital for appropriate treatment strategies and improving patient outcomes in neuro-oncology.

Improved FCM has the degree of membership, non-membership, and hesitation degree [63,73,74]. By addressing the degree of hesitancy throughout the membership function, the improved fuzzy c-mean technique is employed to overcome uncertainty issues [75,76,77]. Traditional clustering techniques cannot handle issues, including outliers and noisy data. To tackle those issues, we took an improved approach. The proposed segmentation model achieved accuracy, precision, and recall, of 98.47%, 98.59%, 98.74% on the Fig share dataset and 99.42%, 99.75%, 99.28% on the Kaggle dataset, respectively, outperforming the competing algorithms. Consequently, compared to the other models described, the proposed model is more capable of detecting the glioma grade, which is highly desirable in clinical applications.

Multiclass Brain Tumor categorization systems have been created to help medical experts visualize and classify different types of tumors. The primary problems with the present approaches to brain tumor classification include low accuracy, lack of data samples for different grades, and binary categorization. The state-of-the-art methods for classifying brain tumors are limited to just two categories: benign and malignant. This makes it very difficult for medical experts to recommend further testing or treatment. Using high-level data from MRI images, ELM is utilized in this study to identify different classes of tumors. This research may help medical experts make decisions about early diagnosis and possible treatment options. This research presents an enhanced ELM for the multiclass categorization of brain tumors to reduce the number of misclassifications. The proposed algorithm achieved the best accuracy for segmentation and classification when compared to the state-of-the-art models.

The challenges for the proposed algorithms are segmenting the tumor region incorrectly due to artifacts and requiring extra time to process due to their computational complexity. The following are the main limitations of this study: (a) The technique was applied to small MRI datasets. To further accomplish even more effective brain tumor segmentation, we will employ large datasets of brain imaging in the future. (b) Further study is needed to make the suggested classification technique compatible with smartphone applications, as it is intended for desktop use only. In future studies, we will examine how to incorporate additional features that can enhance detection and accuracy. Furthermore, it will examine how to shorten the computation time and alter the complexity of the proposed Improved FCM algorithm.

## 6. Conclusions

This study presents a brain tumor detection and classification approach using the improved fuzzy c-means and ensemble learning machine. The proposed FCM minimizes the uncertainty issues while segmenting the MRI images and reduces the computational complexity for identifying the lesion details. Evaluate the present model performance with performance metrics accuracy, precision, and recall, with segmentation values 98.47%, 98.59%, and 98.74% on the Fig share dataset and 99.42%, 99.75%, and 99.28% on the Kaggle dataset, respectively. The proposed model improved brain tumor diagnosis with faster accurate and fast detection times by extracting the color, shape, and texture features. The improved ELM model improves the classification performance. We will implement the proposed model to evaluate the large MRI datasets. We will examine the deep learning models to enhance the model further.

## Figures and Tables

**Table 1:** Class-wise MRI tumor distribution.

Class	Fig Share	Kaggle
meningioma	708	306
Pituitary	930	300
Glioma	1426	300
No tumour	-	405

**Table 2:** Extracted features.

Feature	Definition	Equation
Standard deviation (SD)	Determines the spread of data	$\sqrt{\sum_{k=0}^{W-1}{\left(k-\bar{k}\right)}^{2}p\left(k\right)}$
Skewness	Finds the symmetry of the possibility distribution	$\frac{1}{S^{3}}\sum_{k=0}^{W-1}{\left(k-\bar{k}\right)}^{3}p\left(k\right)$
Energy	Determines the spread of pixel values	$\sum_{k=0}^{W-1}{\left(P(k)\right)}^{2}$
Entropy	Find the data needed to code the data	−$\sum_{k=0}^{W-1}\left[P(k)\log_{2}p\left(k\right)\right]$
Kurtosis	Determines the probability distribution	$\frac{1}{S^{4}}\sum_{k=0}^{W-1}{\left(k-\bar{k}\right)}^{4}p\left(k\right)$
Contrast	Determines local fluctuations	$\sum_{k,l=0}^{W-1}{P_{kl}\left(k-l\right)}^{2}$
Correlation	Determines the joint probability	$\sum_{k,l=0}^{W-1}P_{kl}\frac{\left(k-M\right)\left(l-M\right)}{S^{4}}$
Energy	Determine the sum of the squared pixel values	$-\sum_{k=0}^{W-1}\left[P(k)\log_{2}p\left(k\right)\right]$
Homogeneity	Determines local uniformity	$\sum_{k,l=0}^{W-1}\frac{P_{kl}}{1+{\left(k-l\right)}^{2}}$
Busyness	Determines changes in the neighbouring pixels	$\frac{\sum_{k=0}^{T_{k}}p\left(k\right)q\left(l\right)}{\sum_{k=0}^{T_{k}}\sum_{k=0}^{T_{k}}|kp\left(k\right)-lq\left(l\right)|}$ $where\;p\left(k\right)\neq 0,\;q\left(l\right)\neq 0$
Strength	Determines the primitives of the brain image	$\frac{\sum_{k=1}^{t_{k}}\sum_{k}^{T_{k}}\left[P\left(k\right)\right]{\left(k-1\right)}^{2}}{\sum_{k=1}^{t_{k}}p\left(k\right)}$ $where\;p\left(k\right)\neq 0$

**Table 3:** Comparison of classifier accuracy for Fig Share dataset.

Classifier	Accuracy	Precision	Recall	Class
Proposed	98.56	99.14	99.25	No Tumor
	98.24	98.66	99.19	Glioma
	98.50	99.14	99.10	Meningioma
	98.56	97.43	97.43	Pituitary
Alanazi et al. [55] (2022)	97.86	98.49	98.33	No Tumor
	97.31	98.17	99.14	Glioma
	97.59	97.33	98.64	Meningioma
	97.45	98.51	99.15	Pituitary
Zahoor et al. [56] (2022)	98.31	97.22	89.74	No Tumor
	97.86	98.49	98.30	Glioma
	97.09	98.62	99.16	Meningioma
	98.09	94.59	94.59	Pituitary
Ait Amou et al. et al. [57] (2022)	94.57	98.00	98.50	No Tumor
	95.85	92.10	89.74	Glioma
	95.45	98.24	94.91	Meningioma
	96.76	98.61	98.89	Pituitary
Kirbayi et al. [58] (2022)	94.89	95.90	94.59	No Tumor
	95.04	97.51	98.98	Glioma
	94.89	91.89	87.17	Meningioma
	97.17	96.49	93.22	Pituitary
Poonguzhali et al. [59] (2023)	93.53	97.45	91.42	Pituitary
	92.45	95.35	90.35	No Tumor
	95.35	88.45	96.56	Glioma
	95.45	90.35	97.45	Meningioma
Rahman et al. [60] (2023)	97.34	98.02	95.67	Pituitary
	97.57	98.11	95.78	No Tumor
	97.56	97.13	96.84	Glioma
	97.37	97.24	97.47	Meningioma
Malla et al. [61] (2023)	98.35	97.36	97.25	Pituitary
	98.64	97.46	97.19	No Tumor
	98.50	98.14	99.01	Glioma
	98.36	97.43	97.43	Meningioma

**Table 4:** Comparison of classifier accuracy for Kaggle dataset.

Classifier	Accuracy	Precision	Recall	Class
Proposed	99.37	99.84	99.57	No Tumor
	99.24	99.74	99.85	Glioma
	99.50	99.85	99.84	Meningioma
	99.56	99.57	97.85	Pituitary
Saeedi S et al. [41]	96.46	97.57	98.54	No Tumor
	96.75	97.14	98.36	Glioma
	96.84	97.47	98.27	Meningioma
	96.85	97.78	98.73	Pituitary
Kalam R et al. [42]	97.35	98.46	98.68	No Tumor
	97.15	98.73	98.74	Glioma
	97.36	98.27	98.37	Meningioma
	97.37	98.21	98.62	Pituitary
Mahmud MI et al. [62]	93.53	94.36	95.63	No Tumor
	93.14	94.62	95.63	Glioma
	93.62	94.52	94.63	Meningioma
	93.52	94.26	95.62	Pituitary
Woźniak M et al. [63]	96.46	95.45	95.46	No Tumor
	96.46	95.63	95.74	Glioma
	96.73	95.36	95.47	Meningioma
	96.13	95.63	95.37	Pituitary
Reyes D et al. [64]	97.03	98.17	98.67	No Tumor
	97.19	98.31	98.91	Glioma
	97.25	98.25	98.89	Meningioma
	97.26	98.37	98.59	Pituitary

## Data Availability

The data presented in this study are available on request from the corresponding author.
